# Fecal and Circulating Biomarkers for the Non-Invasive Assessment of Intestinal Permeability

**DOI:** 10.3390/diagnostics13111976

**Published:** 2023-06-05

**Authors:** Nuria Perez-Diaz-del-Campo, Gabriele Castelnuovo, Davide Giuseppe Ribaldone, Gian Paolo Caviglia

**Affiliations:** Department of Medical Sciences, University of Turin, 10126 Turin, Italy; nuria.perezdiazdelcampo@unito.it (N.P.-D.-d.-C.); gabriele.castelnuovo@unito.it (G.C.); davidegiuseppe.ribaldone@unito.it (D.G.R.)

**Keywords:** intestinal barrier, non-invasive biomarkers, intestinal permeability, tight junctions

## Abstract

The study of intestinal permeability is gaining growing interest due to its relevance in the onset and progression of several gastrointestinal and non-gastrointestinal diseases. Though the involvement of impaired intestinal permeability in the pathophysiology of such diseases is recognized, there is currently a need to identify non-invasive biomarkers or tools that are able to accurately detect alterations in intestinal barrier integrity. On the one hand, promising results have been reported for novel in vivo methods based on paracellular probes, i.e., methods that can directly assess paracellular permeability and, on the other hand, on fecal and circulating biomarkers able to indirectly assess epithelial barrier integrity and functionality. In this review, we aimed to summarize the current knowledge on the intestinal barrier and epithelial transport pathways and to provide an overview of the methods already available or currently under investigation for the measurement of intestinal permeability.

## 1. Introduction

The concept of the “leaky gut”, also known as increased intestinal permeability (IP), and its association with the development and progression of a plethora of gastrointestinal disorders, such as inflammatory bowel disease (IBD), celiac disease, and irritable bowel syndrome, as well as other diseases, such as diabetes mellitus, asthma, and Alzheimer’s disease, has progressively gained attention from the scientific community [1].

**Figure 1:**
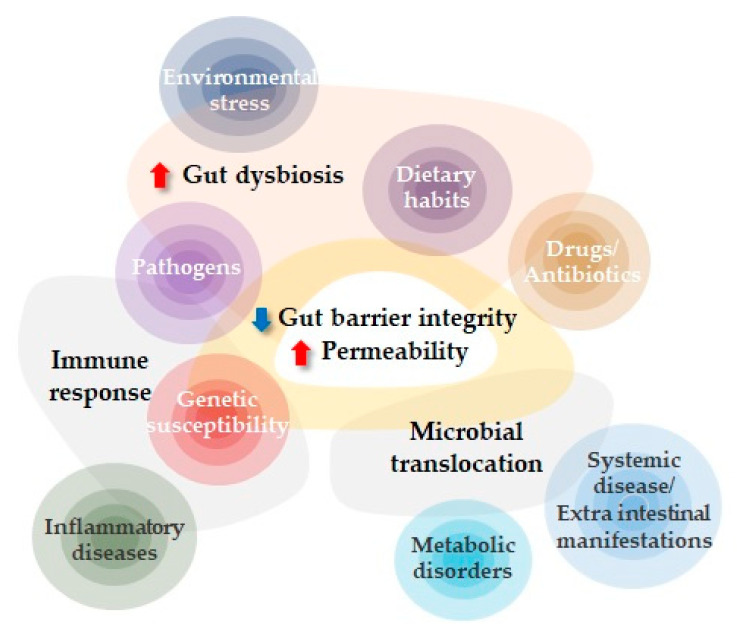
Factors influencing increased intestinal permeability. Genetic susceptibility, environmental factors, dietary habits, and changes in the composition of gut microbiota can affect the intestinal barrier directly or indirectly, inducing dysbiosis that, in turn, can lead to increased intestinal permeability. The loss of intestinal barrier integrity may have a pivotal role in the onset and progression of several gastroenterologic and non-gastroenterologic diseases.

The intestinal barrier is responsible for maintaining gut homeostasis, separating gut microbiota and host immune cells. However, the perturbation of this fine balance by several factors, such as chronic stress, exposure to environmental toxins, such as pesticides, poor diet, or antibiotics, can lead to an increased passage of luminal antigens through the epithelial layer into the underlying mucosa and then to the bloodstream [2]. As a consequence, the translocation of non-self-antigens through the intestinal barrier can induce an abrogation of immune tolerance in genetically susceptible individuals, with the development of chronic inflammation that, in turn, contributes to the perturbation of intestinal barrier integrity (Figure 1). However, studies in humans and mice show that loss of the intestinal barrier alone is not sufficient to initiate disease [3,4]. 

The study of novel methodological approaches and tools for measuring intestinal permeability has rapidly increased in recent years [5,6]. However, how and when to assess intestinal permeability is still a matter of debate. The evaluation of the intestinal barrier in clinical studies is often limited to the assessment of paracellular permeability. Furthermore, not all methodologies used for the assessment of the intestinal barrier are properly validated against the gold standard, i.e., the Ussing chamber, so caution is needed in interpreting these data [7]. In addition, sex-related differences in intestinal permeability have been observed, being lower in women compared to men [3]. In this regard, to better understand the role of increased intestinal permeability in gastrointestinal diseases, it is essential to understand how molecules pass through the intestinal epithelium and how intestinal permeability can be assessed.

## 2. Intestinal Permeability

The “intestinal barrier” is a term that defines the component of the intestine that is responsible for the prevention of harmful substances from passing through the intestinal epithelium (such as bacteria, microorganisms, and their toxins) [8] while allowing the passage of molecules, such as water, electrolytes, and nutrients through the transport systems [9]. To carry out this function, the barrier consists of a heterogeneous structure composed of several elements: mechanical, such as mucus and the epithelial layer; immunological, including IgA, defensins, immune cells, and lymphocytes; and muscular and neurological components [8]. All together, these elements limit the contact between the intestinal epithelium and pathogens, commensal bacteria, and dietary antigens present in the intestinal lumen while allowing the essential absorption of nutrients introduced with food. 

The first element or layer that constitutes the intestinal barrier is the mucus, composed of mucins and fc-gamma-binding proteins, trefoil factor 3, and phospholipid phosphatidylcholine, which confers mechanical protection and the so-called “barrier” and allows the transport of water and macronutrients [10]. The intestinal epithelium, on the other hand, consists of a single layer characterized by the presence of numerous cells that constitute the luminal surface of the small and large intestine, forming a permeable barrier for the passage of food nutrients but that is selective for the passage of harmful molecules [11]. The cells of this epithelium, in order to allow this filtering, are mechanically connected to each other through intracellular protein complexes, such as tight junctions (TJs), adherens junctions, gap junctions, and desmosomes, which allow the regulation of permeability [12]. The epithelium, however, despite being characterized by a single layer, differs according to the intestinal tract. Indeed, while the small intestine is characterized by protrusions called villi and microvilli, which increase the absorptive surface area, and Lieberkühn’s crypts, which are gland-rich invaginations, in the large intestine, the absorptive surface area is smaller due to the absence of this structural organization [10]. 

The term intestinal barrier should not be confused with the term intestinal permeability. While the term intestinal barrier emphasizes the protective component of the gut, intestinal permeability is the functional characteristic of the intestinal barrier, which represents the passage of molecules through the intestinal epithelium and which can be measured by analyzing the flow rate through the intestinal wall either as a whole or in components of the wall of defined molecules [11]. Intestinal permeability can be defined as a diagnostic measure of intestinal barrier integrity and is frequently used for the detection of intestinal mucosal lesions [13]. Interestingly, intestinal permeability can be distinguished between normal, i.e., a stable permeability with no signs of alteration, inflammation, or intoxication, and altered intestinal permeability, i.e., a permeability that leads to the loss of its functional capacity, intestinal homeostasis, and disease [8]. 

Intestinal permeability is highly sensitive to several physiological factors, which can induce an increase in the systemic circulation of bacteria and endotoxins. These factors include damage or alteration to the intestinal barrier layers, as well as changes in the bacterial composition toward a dysbiotic pattern of the gut microbiota [8]. In this regard, factors such as psychological disturbances and lifestyle habits as dietary behaviors based on the Western diet should also be taken into account when assessing the integrity of the intestinal barrier and intestinal permeability, as they may alter it [14,15].

## 3. Epithelial Routes of Transports

**Figure 2:**
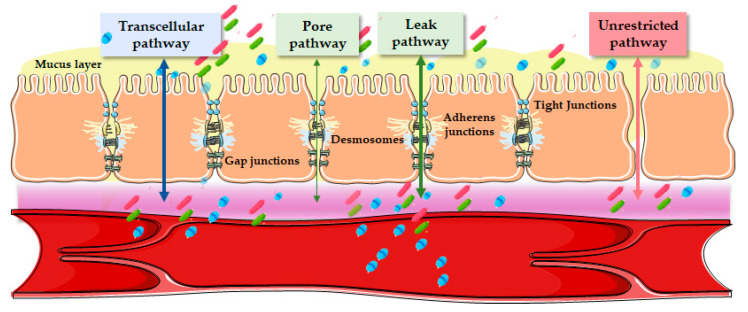
Intestinal barrier and passage routes across the epithelium. There are two main pathways that allow the passage of molecules through the intestinal barrier: the transcellular pathway (used by smaller hydrophilic and lipophilic solutes) and the paracellular pathway (used by larger hydrophilic solutes). Moreover, three different types of paracellular permeability are distinguished, two of which are regulated by tight junctions and are known as the pore pathway and the leak pathway. The last and third pathway is called the “unrestricted pathway”, which is independent of tight junctions and describes flux at sites of epithelial damage.

There are two routes by which the above-mentioned passage through the intestinal barrier can occur, which are the transcellular, involving receptor-mediated transport and diffusion, and the paracellular route, which occurs between two adjacent cells managed by intercellular junctions (Figure 2) [7].

### 3.1. Transcellular Pathway

This pathway is the preferred route for the absorption of hydrophilic and lipophilic compounds, as well as nutrients requiring active transporters and energy, such as vitamins, amino acids, and sugars. These transporters, which can be found on the lateral and apical side of the cell membrane, are selective in terms of charge and molecule size. It is important to emphasize that large molecules, such as proteins, can be absorbed via endocytosis, and are delivered to the lysosomal compartment and degraded to non-immunogenic peptides. The overall process of transcytosis, i.e., active transport through the cytoplasm, serves for the surveillance of antigens in the gastrointestinal tract [16]. Endocytosis and transcytosis have barrier regulatory functions and thus represent manipulable pathways for microbes to enter the host [5].

### 3.2. Paracellular Pathway

In contrast to the transcellular, the paracellular is a less selective pathway utilized by water, ions, and hydrophilic molecules ≤600 Da in vivo to ≤10 kDa in vitro in cell lines [17]. In this regard, this passage occurs passively through intercellular spaces and tight junctions of intestinal cells via diffusion, electrodiffusion, and osmosis according to the gradient generated by the transcellular pathway, thus utilizing size and charge selectivity within the tight junction permeability barrier [5]. The paracellular flow is subjected to regulation by an apical protein complex, consisting of tight junctions, desmosomes, adherens junctions, and gap junctions [18]. However, there is no evidence that the latter two play a primary role in regulating this process [19]. Adherens junctions and desmosomes, on the other hand, are tightly bound between epithelial cells and allow intercellular communication without regulating paracellular permeability, which is instead regulated by the tight junctions surrounding the apical portion of the epithelial cells and which represent a barrier for harmful molecules and the selective passage of solutes and water [10,20]. 

Three different types of paracellular permeability can be distinguished, where two of these three, regulated by tight junctions, define intestinal permeability and are referred to as the leak and pore pathways [21]. The pore pathway, regulated by claudin, is a highly selective route that mediates the movement of ions and small solutes, whereas the leak pathway, which mediates the movement of large solutes, is regulated by occludin, Zonula occludens (ZO)-1, and myosin light chain kinase [22,23]. The third pathway, independent of tight junction regulation, is called the “unrestricted pathway”; it allows antigens access to the lamina propria as it is highly permissive with respect to size and solute charge and is associated with apoptotic leakage in pathological states [23]. During the pathogenesis of the disease, there is initially an increase in permeability through the leak and pore pathways as a result of immune activation of the mucosa, with the production of the tumor necrosis factor (TNF) and interleukin (IL)-13, and subsequently, with disease progression, epithelial apoptosis and permeability through the unrestricted pathway occurs [23]. 

## 4. Non-Invasive In Vivo Assessment of Intestinal Permeability 

For the use and consideration of the “leaky gut” in clinical practice, it is essential to develop and use accurate biomarkers or tools. Several methods are available to assess barrier integrity and permeability, and their selection depends on factors such as the experimental set-up (ex vivo, in vitro, or in vivo), the species (human or animal), the type of marker molecules used (ions or carbohydrates, macromolecules, antigens, bacterial products, or bacteria), and the biological compartments used for the measurement of marker molecules (peripheral blood, portal vein blood, or urine) [8].

As our understanding of the molecular interactions of transporters progresses, the Ussing chamber methodology will continue to provide a “gold standard” in the application of this knowledge. This ex vivo approach is widely used in human and animal studies and allows the assessment of active ion transport across the short-circuit current [24]. Active ion transport generates a potential difference across the epithelium, which is assessed using two voltage electrodes placed as close as possible to the tissue/epithelium [25]. The spontaneous voltage is canceled by injecting a countercurrent called the short-circuit current, which accurately quantifies the net ion transport through the epithelium. In this section, we will address different in vivo methods for assessing intestinal permeability (Table 1). It is important to note that environmental features, such as dietary intake, circadian rhythm [26], or stress [27], can influence intestinal permeability. 

### 4.1. Direct Assessment Using Paracellular Probes

The first techniques used to investigate the integrity of the intestinal barrier function were in vivo permeability assays [5]. This methodology involves the simultaneous use of small-pore-sized markers (5–8 Å) and large-pore-sized markers (9.5–11 Å) that are orally ingested, absorbed in the gastrointestinal tract, and excreted in the urine. Intestinal permeability is calculated as the ratio of the passage of the large-pore marker to the small-pore marker, adjusting for intra-individual confounding factors to obtain a more accurate result [29].

The most widely used probes are small-pore markers, polyethylene glycols (PEG) 400 Da, and monosaccharides (mannitol and rhamnose) as well as the PEG with a molecular weight of approximately 1000 Da [29], or the ^51^Chromium-ethylenediaminetetraacetic acid (^51^Cr-EDTA). In addition, disaccharides, such as lactulose, sucrose, or cellobiose, have been used as markers of gastroduodenal patency [30]. Specifically, the lactulose/mannitol (LAMA) ratio can be interpreted as a measure of leakage pathway permeability and epithelial damage normalized to the surface of the small intestine [31]. Lactulose is large and can only cross the barrier through the leakage pathway or in areas of epithelial damage; thus, it can be considered a marker of barrier integrity [32]. In contrast, mannitol, which is three-fold smaller, crosses the pore pathway and can be considered a measure of surface area. Over the years, the technique has evolved to become increasingly accurate and specific. Studies now use a multi-sugar test (including five different sugar probes: sucrose, lactulose, l-rhamnose, erythritol, and sucralose), which has been shown to provide a more physiological setting for permeability analysis due to its reduced oral dose of lactulose [29]. Importantly, the ratio of erythritol to sucralose is used as an assessment of colonic permeability, whereas lactulose and mannitol are degraded by colonic bacteria making them of little use as a measure of colonic permeability [1].

Both ex vivo and direct in vivo tests are considered reliable for the assessment of intestinal permeability; however, they have some important limitations, such as the lack of standardization, they cannot be performed retrospectively, they are time-consuming and still have limited validity, which is based on the uncertainty of the proposed normal values [33].

### 4.2. Indirect Assessment Using Serum Biomarkers

Over the last decade, particular efforts have been made to identify reliable biomarkers capable of assessing intestinal permeability in blood and, in some cases, in feces. One of the first proteins identified with promising results was zonulin (47 kDa), an acute phase reaction protein that controls intestinal permeability by inducing the disassembly of tight junctions; zonulin has been suggested as a biomarker of intestinal permeability, which can be measured in both blood and fecal samples [10]. In this regard, an intervention study (control diet vs. polyphenol-rich diet) conducted in 66 subjects (aged ≥ 60 years) with increased intestinal permeability based on the measurement of serum zonulin levels showed that zonulin reduction was greater among subjects with a higher body mass index and with insulin resistance at baseline, which demonstrated the close interplay between intestinal permeability and metabolic features [34]. However, recent studies have noted that the current commercially available assays do not detect only zonulin (prehaptoglobin-2) but rather quantify haptoglobin and complement factor C3 levels [35,36]. Similarly, in a study using colonic biopsies from 32 irritable bowel syndrome (IBS) patients and 15 healthy controls, increased colonic paracellular permeability correlated positively with zonulin values in biopsy lysates but negatively with plasma zonulin [37]. In addition, genotyping revealed the unspecificity of the zonulin kit, as all prehaptoglobin 2 non-producers had detectable levels. Therefore, zonulin levels, as a marker of barrier integrity, should be interpreted with caution. 

Furthermore, TJs include several junctional molecules, such as occludin and claudin [38]. In humans, at least 27 subtypes of claudins have been identified that are expressed in an organ-specific manner and regulate the tissue-specific physiological functions of TJs [20]. The examination of claudins in the intestinal context revealed that the absence of intestine-specific claudin-7 (CLDN7) resulted in intestinal inflammation three weeks after birth, together with an elevation of paracellular permeability at two weeks after birth [39]. Similarly, intestine-specific CLDN7 deficiency also increased paracellular permeability for N-formyl-L-methionyl-L-leucyl-L-leucyl-L-leucyl-L-phenylalanine pFlux, a major bacterial product, and subsequently initiated colonic inflammation. On the other hand, occludin is an integral membrane protein that localizes in epithelial and endothelial cells, whose overexpression increases the complexity of the TJ filament network and reinforces the barrier function [40]. It has been observed that twelve weeks of feeding mice with glucose- and fructose-rich diets induced endotoxemia and increased IP to fluorescein isothiocyanate-labeled dextran, an epithelial TJ functional marker [41]. Furthermore, the diets resulted in reduced occludin and ZO-1 values and higher concentrations of inflammation cytokines, including TNF-α and IL-1β, in the colon.

Serum levels of lipopolysaccharide (LPS) are a component of the outer membranes of most Gram-negative bacteria and have also been implicated as a potential marker of bacterial translocation [42]. When the barrier is breached, bacterial endotoxins, such as LPS, can be transferred far into the circulatory system producing toxicity, which has been linked to several diseases [43]. In this regard, a high-fat diet has also been shown to temporarily increase blood levels of LPS in healthy individuals [44]. Furthermore, a study in vitro using human-colon-derived polarized epithelial cell monolayers (Caco-2) demonstrated that species-specific LPS can differentially modulate TLR4-induced inflammation and intestinal epithelial permeability, providing a new concept of serotype-specific epithelial injury and maintenance of the epithelial barrier function in the “leaky gut” [45]. Concerning the methodologies used for the detection of LPS in blood, they have shown, in some cases, inaccurate and contradictory data [46]. In fact, LPS levels have been found to vary considerably according to the method adopted and individuals [47]. Plasma LPS levels should, therefore, be interpreted prudently when attempting to measure a disrupted intestinal barrier, and their combination with other indicators of IP is recommended. As the measurement and interpretation of LPS are complicated, LPS-binding protein (LBP) has become interesting as a marker of the immune reaction to LPS and, thus, as an indirect endotoxemia marker. LBP, an acute-phase protein, is produced by hepatocytes, adipose tissue, and intestinal cells and released in the bloodstream [48]. LBP binds to the external membrane of Gram-negative bacteria and is recognized as a marker of endotoxemia and a biomarker of IP [49]. Moreover, a study aiming to validate six potential biomarkers of intestinal permeability (albumin, calprotectin, and zonulin measured in feces, as well as intestinal fatty-acid-binding protein, LBP, and zonulin measured in plasma) versus the established LAMA test showed plasma LBP as a promising biomarker of intestinal permeability in adults and fecal zonulin as a potential biomarker in overweight and obese individuals [13].

Fatty-acid-binding intestinal proteins (FABPs) are cytosolic proteins of approximately 15 kDa that bind and transport fatty acids and are released into the intestinal lumen upon injury [50]. There are several types of FABPs with different functions depending on the tissue in which they are found. In intestinal enterocytes, mainly both liver-type (L-FABP and FABP1) and intestinal-type (I-FABP and FABP2) fatty-acid-binding proteins are expressed [51]. In addition, ileal lipid- or bile-acid-binding protein (ILBP or BABP and FABP6) is present in the distal ileum, where it has a high affinity for bile acid binding in contrast to the other FABPs present in the intestine [51]. L-FABP and I-FABP exhibit a strong attraction to long-chain fatty acids, suggesting their involvement in intestinal lipid metabolism [52]. A study conducted on humans revealed an association between elevated levels of I-FABP in patients with diarrhea-predominant IBD and an increased small intestinal permeability, which was evaluated through the urine lactulose/mannitol ratio [53]. Therefore, I-FABP appears to be a potential biomarker of intestinal barrier dysfunction.

Citrulline is a non-protein amino acid produced primarily by small intestinal enterocytes with glutamine as a precursor [54]. Serum citrulline has emerged as a valuable biomarker for assessing the mass and surface area of the small intestine. A systematic review showed that there is a negative correlation between citrulline levels and the severity of intestinal disease, such as celiac disease and Crohn’s disease [55]. This suggests that a decrease in small intestinal epithelial mass contributes to increased intestinal permeability. Notably, patients undergoing haemopoietic stem cell transplantation due to mucositis in the oral and gastrointestinal regions exhibited a decline in circulating citrulline levels due to the loss of epithelial mass [56]. Another study compared the citrulline assay to the in vivo multiple sugars assay and found that the former demonstrated higher specificity and sensitivity in evaluating small bowel permeability among patients undergoing myeloablative therapy [57]. It is important to note that citrulline, being a non-protein amino acid, is influenced by food absorption [58]. However, although watermelon contains a small amount of citrulline (1 g citrulline/780 g), increasing watermelon intake over a three-week period did not result in an increase in plasma citrulline concentration [59]. Therefore, caution is necessary when interpreting citrulline levels as an indicator of intestinal permeability.

Soluble CD14 (sCD14) is considered a marker of monocyte activation. The binding of LPS to CD14 has been shown to activate the innate immune system [60]. Importantly, in a case-control study including 74 subjects who died, 120 of whom developed cardiovascular disease and 81 of whom developed acquired immunodeficiency syndrome during the Strategies for Management of Anti-Retroviral Therapy study with matched control subjects, it was shown that sCD14 was an independent predictor of mortality in human immunodeficiency virus infection [61].

Glucagon-like peptide (GLP)-2 is a cleavage product of glucagon located mainly in the gastrointestinal tract and central nervous system [62]. GLP-2 is involved in the maintenance of growth [63] and in intestinal mucosal integrity, gastric motility, and nutrient absorption [64]. In a study in obese mice fed with a prebiotic diet, changes in the microbiota and increases in endogenous GLP-2 production were observed [65]. In particular, there was an increase in bifidobacterium and lactobacillus species and a reduction in plasma LPS levels and, consequently, an improvement in intestinal barrier functions via a GLP-2-dependent mechanism.

Glycoprotein VI (GPVI), the platelet receptor for the immunoreceptor tyrosine activator motif for collagen, plays a prominent role in vascular integrity in animal models of inflammation and sepsis [66]. Using an in vivo model of autoimmune arthritis, the presence of endothelial gaps in the inflamed synovial membrane was confirmed. Surprisingly, patency in inflamed joints was abrogated if platelets were absent [67]. Indeed, GPVI has been implicated in the recruitment of platelets and leukocytes to the inflamed vascular wall, the regulation of vascular permeability and leukocyte activation, and in the prevention of inflammatory hemorrhage [66].

Calprotectin, lactoferrin, and elastase are neutrophil-derived proteins present in feces, with calprotectin being the most detectable due to its resistance to proteolytic degradation and its stability in feces stored at room temperature for at least seven days [13]. In fact, fecal calprotectin is nowadays used in clinical practice to assess disease activity in the follow-up of patients treated for active IBD. In a case-control study (28 controls and 34 patients with Parkinson’s disease (PD)), calprotectin, alpha-1-antitrypsin, and zonulin were found to be significantly elevated in PD patients compared to age-matched controls [68]. Other proposed markers of intestinal permeability include secretory IgA used in celiac disease or defensins mainly analyzed in IBD patients as markers of intestinal permeability [69]. 

Similarly, when the intestinal barrier is damaged, albumin can pass from the blood vessels into the interstitial space and finally into the intestinal lumen. Therefore, it has been suggested that fecal albumin is a biomarker of intestinal permeability [70]. Alpha(α)-1-antitrypsin (AAT) is a highly abundant serine protease inhibitor found in the bloodstream. Although primarily synthesized in the liver, it is also secreted by different cell types, including macrophages, enterocytes, and Paneth cells [71]. AAT plays a crucial role in protecting tissues against the proteolytic actions of immune cells, especially neutrophils [72]. In addition, AAT levels have been found to correlate with Crohn’s disease activity, and feces may serve as an indicator of disease severity in IBD [73].

Other markers of intestinal inflammation that could be assessed to complement AAT measurements and other in vivo permeability markers include fecal or serum lipocalin-2 (LCN2) and serum amyloid A [74]. LCN2 is produced by various cell types, including myeloid and intestinal epithelial cells, and is elevated in response to a wide variety of pro-inflammatory stimuli [74]. In this regard, in a cohort of 132 patients with IBD, the mucosal LCN2 expressions remained elevated in the rectum of ulcerative colitis and the ileum of Crohn’s disease patients [74]. Similarly, acute-phase protein amyloid A has also been investigated in IBD [75]. It is an acute-phase lipoprotein, which may play an important role in binding and removing cholesterol from inflammation areas [66]. Moreover, it has been shown that T-cell signaling pathways modulated by serum amyloid A proteins may be attractive targets for anti-inflammatory therapies [76].

Finally, short-chain fatty acids (SCFAs), such as acetate, propionate, and butyrate, are the main end products of intestinal microbial fermentation. SCFAs have been shown to improve intestinal epithelial barrier function, restoring damaged epithelium [77]. For example, butyrate has been shown to have anti-inflammatory and regenerative properties, providing symptomatic relief when administered orally to patients suffering from various diseases of the colon [78]. In a cohort of 127 patients with ulcerative colitis, a reduction in butyrate-producing bacteria (R hominis and F prausnitzii) was observed compared to healthy individuals [79]. Both species showed an inverse correlation with disease activity. The absence of butyrate can, therefore, be considered an indirect indication that the intestinal barrier function is compromised.

Overall, ongoing research on the novel biomarkers of intestinal permeability holds significant potential for advances in the field of gut health. Biomarkers related to intestinal permeability might play a key role in assessing the efficacy of therapeutic interventions. The development of non-invasive techniques for biomarker detection, such as blood, urine, or stool tests, together with molecular insights, lifestyle modifications, and dietary interventions, could lead to early diagnosis and continuous monitoring of intestinal disorders and conditions associated with impaired permeability. 

## 5. Conclusions 

Although several gastrointestinal and non-gastrointestinal disorders result in a leaky gut, it is uncertain whether a cause–effect relationship exists between increased intestinal permeability and disease onset and progression; nevertheless, the possibility to evaluate intestinal barrier integrity is crucial to improve patients’ clinical management. Indeed, intestinal barrier healing has been proposed as a potential therapeutic endpoint associated with a favorable prognosis [80]. 

To date, several different non-invasive tests show promising results, although some analytical limitations still need to be solved. Likely, a composite assay able to simultaneously investigate biomarkers with different biological roles, such as TJ structural proteins, in association with proteins involved in TJ regulation, pro-inflammatory cytokines, and bacterial translocation markers might provide a reliable tool to accurately evaluate intestinal barrier integrity. 

## Figures and Tables

**Table 1:** Selection of in vivo permeability assays.

	Method	Human Studies	Animal Models	Expression Site	Biological Sample	Biomarker Levels and Increased IP
Lactulose/mannitol	Dual sugar quantification using mass spectrometry	X	X	Small intestine	Urine	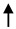
Sucralose	Dual sugar quantification using mass spectrometry	X	(X)	Colon	Urine	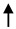
Sucrose	Dual sugar quantification using mass spectrometry	X	(X)	Stomach	Urine	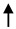
PEG 4000/400 kDa	Quantification using mass spectrometry	X	(X)	Whole intestine	Urine	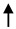
^51^Cr-EDTA	^51^Cr-EDTA radioisotope activity	X	X	Whole intestine	Urine	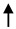
Zonulin	ELISA	X	X	Small intestine	Feces/serum	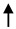
LPS	LAL assay	-	X	Whole intestine	Serum/plasma	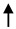
LBP	ELISA	X	X	Whole intestine	Serum/plasma	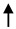
sCD14	ELISA	X	X	All sites	Serum/plasma	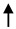
FABP	ELISA	X	X	All sites	Serum/plasma	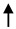
Citrulline	Mass spectrometry	X	X	Small intestine	Serum/plasma	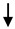
Claudin	ELISA	X	X	All sites	Serum/plasma	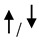 *
Occludin	ELISA	X	X	All sites	Serum/plasma	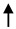
Glycoprotein VI platelet	ELISA	X	X	All sites	Serum/plasma	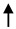
Glucagon-Like Peptide-2	ELISA	-	X	Whole intestine	Serum/plasma	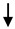
Calprotectin	ELISA	X	X	Whole intestine	Feces	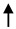
LCN-2	ELISA	X	X	Whole intestine	Feces	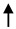
SCFA	Gas/liquid chromatography	X	X	Colon	Feces	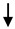

For each biomarker, the X indicate the availability of data in humans and in the animal model. Abbreviations: ^51^Cr-EDTA, 51 chromium-labeled EDTA; FABP, fatty-acid-binding protein; LAL, limulus amebocyte lysate assay; LBP, lipopolysaccharide-binding protein; LCN-2, lipocalin-2; LPS, lipopolysaccharide; PEGs, polyethylene glycols; sCD14, soluble CD14; SCFA, short-chain fatty acid. * Claudin-2 is highly expressed in permeable epithelial tissues, is upregulated in IBD, and promotes inflammation, while down-regulation of claudin-5 and -8 can drastically reduce barrier integrity [28].

## Data Availability

All data are publicly available.
